# Tapping natures rhythm: the role of season in mitochondrial function and genetics in the UK biobank

**DOI:** 10.1186/s40246-025-00743-8

**Published:** 2025-03-29

**Authors:** Anastasios Papadam, Mihail Mihov, Adriana Koller, Hansi Weissensteiner, Klaus Stark, Felix Grassmann

**Affiliations:** 1 Institute of Medical Science, University of Aberdeen, Aberdeen, UK; 2 Institute of Genetic Epidemiology, Medical University of Innsbruck, Innsbruck, Austria; 3 Department of Genetic Epidemiology, University of Regensburg, Regensburg, Germany; 4 Institute for Clinical Research and Systems Medicine, Health and Medical University, Potsdam, Germany; 5 Institute for Clinical Research and Systems Medicine, Health and Medical University, Schiffbauergasse 14, 14467 Potsdam, Germany

**Keywords:** Mitochondria, Season, Genomics, Metabolomics, Transcriptomics

## Abstract

**Background:**

Mitochondria are small organelles inside our cells crucial for producing energy and heat, cell signaling, production and degradation of important molecules, as well as cell death. The number of mitochondria in each cell is a marker for mitochondrial function, which generally declines with increasing age. However, we found that there is also a considerable seasonal variation of mitochondrial abundance, which warrants further research.

**Methods:**

We leveraged data from individuals participating in the UK Biobank study and computed their mitochondrial abundance from Exome sequencing reads mapping to the mitochondrial genome. The seasonal effect was modelled as a sine-cosine function across the year and changes in amplitude, acrophase and displacement of mitochondrial abundance due to various demographic, lifestyle, genetic, proteomic, and metabolomic markers were investigated with multivariate regression.

**Results:**

We found that mitochondrial DNA (mtDNA) abundance was higher in winter than in summer. This difference is related to advanced age, a higher BMI and smoking behavior which resulted in a reduced amplitude of mtDNA abundance. A higher education reduced the acrophase (i.e., shifted the distribution to earlier in the year) and a higher BMI and lack of physical activity led to a later acrophase. Generally, increased immune cell count resulted in lower amplitude, and an increased platelet and lymphocyte count was found to increase the acrophase. Importantly, a reduced seasonal amplitude was associated with increased risk for cardiovascular, digestive, genitourinary, and respiratory diseases as well as all-cause mortality. Most of the metabolomic and proteomic markers were associated with mtDNA displacement (i.e., increase of the baseline level) but not acrophase or amplitude. Similarly, we found that there are multiple genetic variants influencing displacement, but none reached genome-wide significance when investigating acrophase or amplitude.

**Conclusion:**

Seasonal variation of mtDNA abundance is influenced by environmental, lifestyle and immune parameters. Differences in the seasonal oscillation of mitochondrial abundance could potentially explain discrepancies of previous associations results and might be useful to improve future risk prediction.

**Supplementary Information:**

The online version contains supplementary material available at 10.1186/s40246-025-00743-8.

## Introduction

**Fig. 1:**
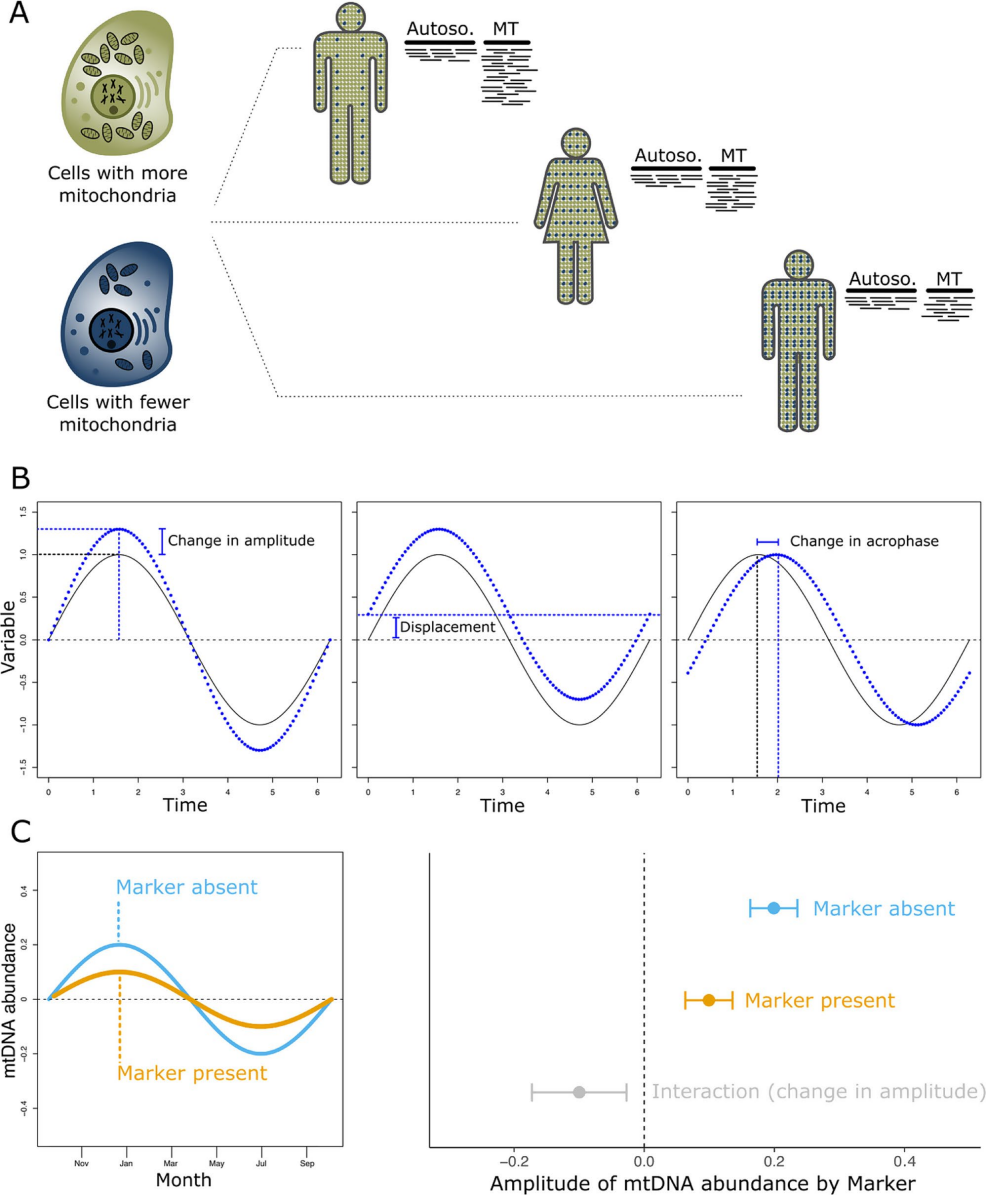
Overview of the methodology used in this study. **A**: The amount of mitochondrial DNA in circulating blood cells was determined from the Exome sequencing reads mapping to the autosome and the mitochondria. Less mitochondrial DNA (mtDNA) abundance results in fewer MT specific reads compared to autosomal reads. **B**: Environmental, lifestyle, genetic or metabolomic markers can influence three aspects of the seasonal pattern in mtDNA abundance: A change in amplitude would increase the difference between the minimum and maximum value observed across each year. In contrast, markers influencing the acrophase would result in the whole seasonal pattern shifted by a certain amount of time. Displacement describes the effect that the baseline level is shifted while not influencing the overall cosine pattern. This is equivalent to looking for markers that influence overall mtDNA levels, as was done in previous studies. **C**: We used linear regression models to investigate the effects of markers on amplitude or acrophase. In case a marker reduces the amplitude, the interaction term (grey) in the regression model would be negative and quantifies the difference between the amplitude observed with (orange) and without the marker present (blue)

Mitochondria, in addition to generating energy through oxidative processes, are involved in heat production, iron storage, apoptosis, intra- and extra-cellular cell signaling, biosynthesis and degradation of important metabolites as well as processing of therapeutic agents. Depending on the tissue, cells host a dynamic range of multiple mitochondria which in turn contain multiple copies of a tiny circular genome (~ 16,569 base pairs). The human mitochondrial proteome consists of more than 1,100 proteins, of which only 13 are encoded in the mitochondrial genome [1]. In contemporary human populations, several haplogroups are defined by ancestral and stable mutations in the MT genome. Those haplogroups are derived from adaptation to different geographic areas under distinctive selection pressure [2] and may also influence the abundance of mitochondrial DNA (mtDNA) [3–5], although there is conflicting evidence [6, 7]. Notably, different haplogroups have been reported to be associated with diseases as well as longevity [8, 9] and as such contribute to phenotypic variety within human populations. However, haplogroups do not appear to influence mtDNA abundance directly [10] when accounting for known confounding factors. Mitochondria rely on self-replication and are thus prone to cellular stress in aging and diseases. Hence, mitochondrial dysfunction and a reduction in its biogenesis (and thus a reduction in the amount/abundance of mtDNA) are hallmarks of aging [11] and has been associated with most aging-related diseases [12–14] as well as immunological processes [15]. To quantify the amount of mtDNA as a marker of mitochondrial abundance and thus function and health, several methods have been proposed such as quantitative real time PCR (qPCR), digital droplet PCR (ddPCR), high-throughput genotyping arrays or using the sequencing depth of reads aligned to the mitochondrial genome from exome or whole genome sequencing (Fig. 1A). Generally, qPCR, ddPCR and next generation sequencing approaches result in the most accurate estimation of mtDNA content in circulating blood cells.

While the amount of mtDNA and thus the number of mitochondria in blood cells is slightly stable across an individual’s lifetime (temporal intraclass variation ranging from 38% to 60%) [16, 17], there are considerable differences between individuals, which potentially is an important marker for health or disease. Thus, it is crucial to identify further markers influencing mitochondrial abundance, particularly in light of conflicting association results in previous reports regarding the role of mitochondrial DNA abundance in blood on disease risk and outcomes such as survival or disease progression [18–24]. This was often attributed to the limited effect of mtDNA abundance in blood on the diseases or to the method of quantification and/or normalization. Nevertheless, we argue that a different mechanism could be responsible for the lack of associations or conflicting results: We found that the amount of mitochondrial DNA changes drastically across the year, a phenomenon previously observed in hibernating mammals [25–27]. The seasonal pattern can be approximated according to a sine wave and thus further investigated. There are three main parameters of the sine wave that can be modelled in regression models (Fig. 1B): First, displacement is a vertical shift of the wave and corresponds to marginal effects observed in the regression model. This parameter corresponds to overall mitochondrial DNA abundance as ascertained in previous studies, while adjusting for the presence of the seasonal effect. Second, we can investigate changes in the amplitude of the sine wave, which results in an increased peak or decreased trough (low-point). Finally, changes in the acrophase mean that the whole sine wave is shifted horizontally, thus the peak and trough of the wave are reached earlier or later in the year. To understand the impact of individual demographic, lifestyle, metabolomic and proteomic markers as well as blood cell counts on those parameters, we studied a large collection of individuals recruited as part of the UK Biobank.

## Methods

### Study population

The current study was conducted using the UK Biobank, which is a prospective cohort of over 500,000 individuals with available genotyping data as well as a rich collection of questionnaire and biomarker data. The genotyping chip UK Biobank Axiom array was used to obtain the genetic information of the UK Biobank participants, which includes 784,256 variants [28]. We included all consenting individuals and removed non-European samples, samples failing genotyping quality control, samples without exome sequencing data available, as well as those having sex chromosomes aneuploidies, resulting in a final dataset of 385,667 samples. An overview of the used sample is found in Table 1.

**Table 1:** Study population

Variable	Females	Males	All
Number of individuals	208,269	177,398	385,667
Mean age at recruitment (S.D) [years]	56.72 (7.92)	57.15 (8.09)	56.92 (8.00)
Mean body mass index (S.D) [kg/m2]	27.05 (5.06)	27.83 (4.17)	27.41 (4.69)
Packyears (S.D) [P/dY]	6.39 (12.87)	11.01 (18.78)	8.48 (15.99)
Ever smoked [%]	40.85	51.25	45.64
Risky alcohol consumption [%]	41.91	56.76	48.74
Lack of physical activity [%]	13.98	15.63	14.74
University degree [%]	29.27	32.30	30.66
Mean Frailty Index (S.D)	12.04 (6.73)	11.47 (6.64)	11.78 (6.70)
Mean eGFR [mL/min/1.73m2] (S.D)	90.89 (13.66)	89.73 (12.80)	90.36 (13.28)

## Ascertainment of environmental, lifestyle and immunological factors

BMI was ascertained at the recruitment center at the time of the baseline exam and coded as a linear variable. Missing BMI values (2% missingness) were imputed to the median to improve power. We used self-reported questionnaire data to establish variables for smoking status (ever smoked vs. never smoked or missing, 0.4% missingness), pack-years (in years smoked at least one pack per day), alcohol consumption (more than three drinks a week vs. less or missing, 3.8% missingness), lack of physical activity (less than moderate/vigorous/walking recommendation vs. more or missing, 19.9% missingness) and education (university degree vs. none or missing, 18.9% missingness). For never-smokers, we set pack-years to 0 and imputed the remaining to the median of the respective smoking group (i.e., past or current smokers, overall missingness: 15.5%). In addition to those factors, we included markers related to white and red blood cell counts (field 100081), blood chemistry (field 17518) and markers measured in urine (field 100083). The frailty index was computed as previously described [29]. Briefly, the total number of self-reported disease and ailment was ascertained from questionnaire and normalized to the total number of possible answers, resulting a frailty index representing the percentage of self-reported ailments. To compute the estimated glomerular filtration rate (eGFR), we used the CKD-EPI creatinine equation [30]. First, the molar creatinine levels obtained from the UK Biobank (field 30700) were converted to mg/dl and then used in the equation separately for males and females. Missing values in the eGFR were imputed to the median (6.5% missingness).

## NMR metabolomics

We used data from the initial release of the NMR metabolomics (field 220) to assess their association with mitochondrial seasonal variation. Values retrieved for all data fields in category 220 were processed with the *ukbnmr* package [31] in R. Briefly, the decoded data was loaded into R and the *remove_technical_variation* was used to remove technical variation due to shipment plates, time between sample preparation and sample measurement, and analysis spectrometer. The resulting values were log2 transformed and then scaled to have a mean of 0 and a standard deviation of 1 for use in the regression analyses. Association results were plotted as a mirrored manhattan plot using *ggplot2* [32].

## Proteomic analyses

Relative abundance measures of 2923 proteins in plasma were retrieved for around 50,000 UKB participants from data field 1838. The data was transformed to long format with the *dplyr* package in R and missing values were imputed with the imputePCA function from the *missMDA* package (mean missingness: 10,3%, S.D. 7.4%). The abundance of the proteins is given as NPX values (Normalized Protein eXpression), which is in log2 scale. The NPX values were used as exposure in the association testing and the resulting P-values are plotted in a manhattan plot.

## MtDNA abundance quantification

The amount of mtDNA relative to the autosomal DNA was quantified using the read depths obtained from exome sequencing reads mapped to the mitochondrial and autosomal genome. Importantly, the exome capture used by the UK Biobank does not enrich for mitochondrial sequences, thus the obtained coverage (on average of 0.66X or roughly 11,000 reads in total) of the mitochondrial genome is due to unspecific fragments bound to the capture beads. This allows for an unbiased quantification of mtDNA abundance, which would potentially be influenced by saturation effects of capture probes and capture efficiency [33]. However, the coverage is too low to compute haplogroups from the reads, therefore we computed the haplogroups from the microarray data with haplogrep [34], as previously described [35]. Next, we used mosdepth [36] to calculate the coverage of the mitochondrial genome in each individual and divided that number by the total number of reads mapped to the genome. Individuals with an mtDNA abundance larger or smaller than 4 times the interquartile range from the median were excluded from the analysis (total number of excluded individuals: 9650). The normalized mtDNA abundance ratio was coded as a linear factor and used as the main outcome or exposure in this study.

### Disease ascertainment from hospital episode spell data

In order to conduct a phenome-wide association study, we extracted all diseases diagnosed (as ICD9 or ICD10 codes) from the hospital episode spell data and mapped those codes to phecodes with the *PheWAS* package [37] implemented in R. The analyses were restricted to incident diseases (i.e., diseases occurring after recruitment), and prevalent cases were removed from the respective disease by setting those patients to missing. We grouped diseases into their major disease groups according to the organ or system affected by the disease: Circulatory, digestive, endocrine, genitourinary musculoskeletal, neurological, and respiratory system as well as neoplasms, mental disorders and autoimmune diseases. We also computed a phenotype that included individuals with any recorded disease (out of the major disease groups) and those with multiple diseases. Finally, we conducted a PheWAS for all diseases occurring at least in 500 individuals during follow-up, adjusted for the above-mentioned covariates.

## Genome- and transcriptome-wide analyses (GWAS and TWAS)

To assess the role of inherited genetics in governing the displacement, amplitude and acrophase of seasonal mtDNA patterns, we used the TOPmed imputed genetic data available for all UKB participants, which passed quality control (see above). We removed variants deviating from Hardy-Weinberg-Equilibrium (*P* < 5*10^− 08^), imputation quality < 0.4, a minor allele frequency below 5% and those missing more than 10% of their genotypes. The resulting summary statistics (one for displacement, amplitude and acrophase) were then used to compute the heritability of the factor using LD Score Regression [38] with standard settings. Similarly, we used the summary statistics to perform a transcriptome-wide association study with TWAS-fusion [39] across whole blood samples from GTEx v6, NTR (Netherlands Twin Registry) and YFS (Young Finns Study). Results were plotted as mirrored manhattan plots using *ggplot2*. We also compiled qq-plots for each GWAS with the *qq* function from the *qqman* package in R [40].

## Seasonal analyses

The main factor we wanted to investigate in this study was the seasonal patterns in mitochondrial abundance. Generally, the seasonal pattern seems to follow a cosine wave function across one year. Thus, we modelled several aspects of that curve. The sine component of season wave (SINW) was computed fromundefined

$$\>SINW = {\rm{sin}}\left( {2*\pi *{{\left( {month - 1} \right)} \over {12}}} \right)$$

where month was the numeric number of the respective month of recruitment of each individual, starting with January as 1 and December as 12. sin() is the sine function implement in R. Similarly, the cosine part (COSW) was computed as

$$\>COSW = {\rm{cos}}\left( {2*\pi *{{\left( {month - 1} \right)} \over {12}}} \right)$$

where cos is the cosine function. Both the sine and cosine component were included in a linear regression model with the mitochondrial abundance as the outcome and sine/cosine values as exposures as well as additional factors for adjustment (see statistical analyses for details). Those two functions effectively capture the sinusoid part of the seasonal pattern and can then be transformed to represent the amplitude and acrophase of the pattern. Briefly, we extended the approach from the *cosinor* package in R to allow the computation of amplitude and acrophase as well as their standard errors also from linear variables [41]. The estimate for the amplitude was computed as

$$\:\text{Amplitude}=\:\surd\:\left(SIN{W}^{2}+COS{W}^{2}\right)$$

And the acrophase estimate was computed as:

$$\:\text{Acrophase}=\text{atan\:}\left(\frac{SINW}{COSW}\right)$$

where atan is the arctangent (inverse tangent). To compute the standard errors for those estimates, the same transformation cannot be easily applied to the standard errors for SINW and COSW. Here, we used the delta method to approximate the standard errors for the estimate from the regression model using a first-order Taylor approximation. Dividing the estimate by the standard error results in a T-Score of each association, which can be used to compute the 95% confidence intervals of the estimate as well as a P-value to assess statistical significance.

The above approach allows us to quantify the sinusoid part in the seasonal pattern depending on the month of recruitment. However, to assess whether this pattern is influenced by other factors such as lifestyle markers (Fig. 1B) requires one additional step. Here, we included an interaction term between the investigated marker and the SINW and COSW variable:

$$\eqalign{& \>y = \>\beta {\>_0} + \>\beta {\>_1}M + \beta {\>_2}COSW + \beta {\>_3}SINW + \cr & \beta {\>_4}M*COSW + \>\beta {\>_5}M*SINW + \ldots \> + \beta {\>_i}{C_i} \cr} $$

Where $\:y$ is the outcome (mtDNA abundance), M donates the marker values, $\:{\beta\:}_{0}$ is the intercept and $\:{\beta\:}_{1}-\:{\beta\:}_{5}$ are the slopes/beta values obtained in the regression analysis. $\:{C}_{i}$ are additional covariates in the model (such recruitment center, genotyping principal components, hour and year of recruitment), with the corresponding slopes ($\:{\beta\:}_{i}$). SINW and COSW are the sine and cosine components, as shown above. The change in amplitude and acrophase (either due to the absence/presence of the marker or per unit of the marker) was then extracted from the interaction term estimate of the regression model ($\:{\beta\:}_{4}$ and $\:{\beta\:}_{5}$, Fig. 1C) as outlined above and the corresponding standard errors were retrieved with a first-order Taylor approximation. Importantly, the marginal effect of the marker ($\:{\beta\:}_{1}$ in the equation above) corresponds to the displacement of the sinusoid curve, i.e., its vertical shift and thus the overall change in mtDNA abundance accounting for the seasonal pattern. Using $\:{\beta\:}_{2}$ and $\:{\beta\:}_{3}$, it is possible to compute the overall amplitude and acrophase of mtDNA across the year, as outlined above.

### Statistical analyses

The association of environmental, lifestyle, immune and red blood cell marker were conducted with linear regression with the mitochondrial abundance quantified from exome sequencing reads as the outcome. For each marker, we included an interaction term between that marker and the SINW and COSW variable as note above (see Fig. 1C). Generally, in addition to SINW and COSW, all models were adjusted for age, sex, baseline BMI, smoking status, education, lack of physical activity, risky alcohol consumption, Vitamin D levels in blood, the hour and year of blood draw, recruitment center, the first ten principal components of ancestry computed from genotyping data, white blood cell and red blood cell count. In order to analyze the impact of blood cell counts on mtDNA abundance seasonal variation and to account for correlation between the markers, we additionally included all investigated blood cell markers in the same model but only computed the interaction with one of the markers at a time. Next, we computed the change in amplitude and acrophase from the resulting slopes of the interaction term. The effect size of the resulting amplitude was normalized to the maximum amplitude (i.e., divided by 0.091 standard deviations), thus representing the change in the amplitude in percent. Acrophase represents the change in the acrophase in percent of a month and was not further transformed. The results of the correlation analyses were visualized with a correlation plot using the *corrplot* function from the *corrplot* package, implemented in R. In the correlation plot, we deemed correlations with an uncorrected P-value of less than 0.05 as statistically significant.

The phenome-wide association study was performed on incident major diseases as retrieved from the phecodes computed by the PheWAS package. The disease status was the main exposure, and we estimated whether individuals that develop a certain disease have a different amplitude, acrophase or displacement of mtDNA across the seasons. The analyses were adjusted for the same variables as above and additionally for lymphocyte and neutrophil count as well as hematocrit. The results were then visualized as a forest plot using *ggplot2*. For the PheWAS including all individual phecodes with at least 500 observations (640 in total), we accounted for multiple testing with a Bonferroni correction for 1920 tests (i.e., 3 times 640 diseases) and considered adjusted P-values below 0.05 as statistically significant.

Similarly, the laboratory marker wide association scan (labWAS) and the proteome-wide association study (PWAS) were conducted with linear regression. Of note, the labWAS was adjusted for the same variables above and also adjusted for total cholesterol to reduce the impact of total cholesterol on the results, which can skew metabolomic results in case the outcome has an association with it (which we found in a previous study [35]). Similarly, to account for residual confounding of technical artifacts, the proteomic analyses were adjusted for the factors mentioned above and, additionally for the first three principal components computed from the protein data. In these two analyses, we considered P-values as significant if they are smaller than 0.0001 (i.e., 0.05/379 laboratory markers) or smaller than 0.000015 (i.e., 0.05/2923 protein markers) in the labWAS and PWAS, respectively.

In the genome-wide association study, we also modelled mitochondrial abundance as the outcome and extracted the change in amplitude and acrophase from the interaction term between the genetic variant and the SINW and COSW variables. The analyses were adjusted for as stated above and, additionally for lymphocyte and neutrophil count as well as hematocrit. We considered P-values for association below 5.00 × 10^− 08^ as genome-wide significant and associations below 5.00 × 10^− 06^ as transcriptome-wide significant.

The survival analysis was conducted with a linear regression model with mitochondrial abundance as the outcome. We assessed whether the censor variable (death from any cause = 1, end of follow-up or lost to follow-up = 0) showed a significant interaction with the amplitude or acrophase as outlined above. The model was adjusted for the same variables as above and, additionally, for the frailty index, length of follow-up, hematocrit, lymphocyte and neutrophil count.

## Results

The summary characteristics of study participants by sex status are listed in Table 1. We computed the abundance of mitochondrial DNA (mtDNA) relative to autosomal DNA from the exome sequencing reads of all participants.

### Mitochondrial DNA abundance is influenced by season

**Fig. 2:**
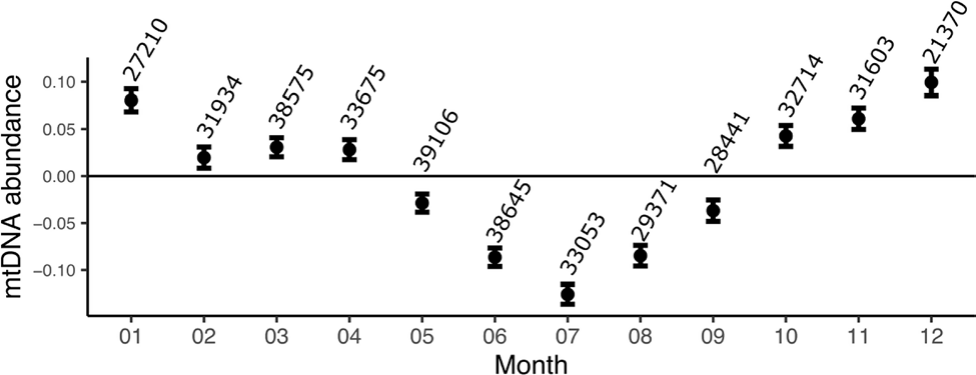
Seasonal variation of mitochondrial DNA abundance in the UK Biobank. The amount of mtDNA in blood cells varies with season in the UK Biobank. The average mtDNA abundance in S.D. from the mean (zero) is depicted as a dot and the borders of the 95% confidence intervals as horizontal bars. Numbers above each estimate indicate the sample size in each month. The seasonal effect was observed overall three years of recruitment and across all recruitment sites and was virtually unchanged when adjusting for most factors known to influence mtDNA abundance

In this study, we found that the mtDNA showed a seasonal pattern (Fig. 1), with peak (crest) abundance reached in winter around winter solstice and the trough (low point) observed in summer (around summer solstice, i.e., 6.7 months into the year). We modelled the change in abundance as a sine (sinusoid) wave and obtained an amplitude of 0.092 standard deviations (95% confidence interval: 0.085; 0.097, P-value = 2.51 × 10^− 189^) and an acrophase of -0.14 (95% CI: -0.078; -0.21, P-value = 1.80 × 10^− 05^), corresponding to roughly one week before new year’s eve. The linear regression model was adjusted for lifestyle factors influencing mtDNA abundance. Additional adjustment for blood cell count, Vitamin D levels, haplogroup (estimated from genotyping arrays), recruitment center and hour of blood draw did not change the observed amplitude or acrophase. Importantly, the size of the amplitude is similar to the difference between males and females (0.086 standard deviations) or to the decline in mitochondrial abundance of around 20 years (0.005 standard deviations per year). The amplitude and acrophase explain around 0.4% of the variation in the mitochondrial abundance observed (Fig. 2).

### Lifestyle, demographic, and blood cell markers influencing the seasonal pattern

**Fig. 3:**
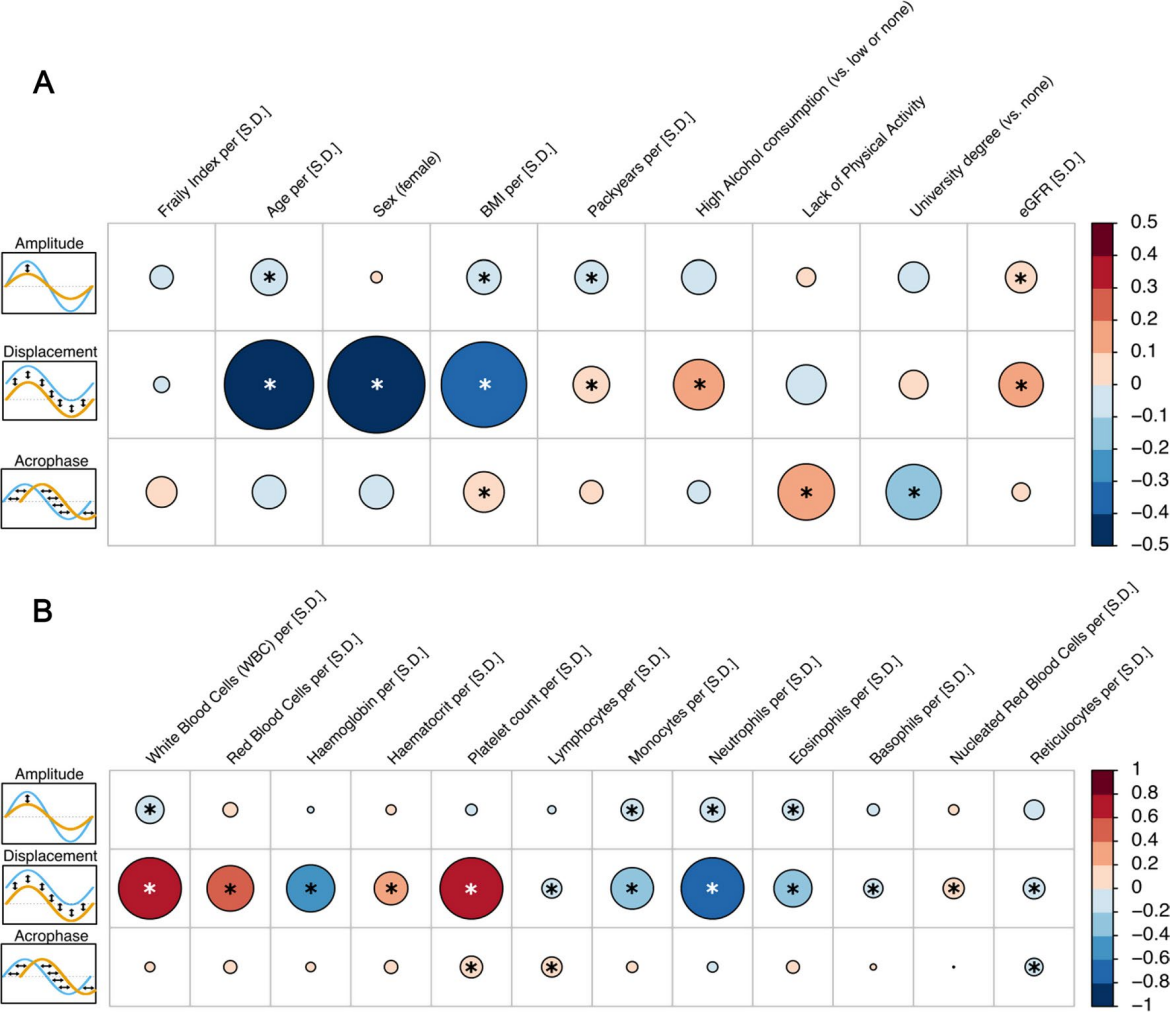
Lifestyle, demographic factors, and blood cell count influencing mtDNA seasonal patterns. The size and color of the circles indicate the effect on the seasonal pattern of mitochondrial abundance. Blue circles indicate that the marker reduced the respective pattern and red increased it. Amplitude designates the effect on the difference between the minimum and maximum values in the seasonal pattern and is given as the fraction of the total amplitude (which is 0.091 standard deviations). Displacement is the vertical shift in the mean value across all seasons (i.e., the marginal effect in the regression model) and is given in % of a standard deviation. The change in the time until the peak in the wave is reached is described by the acrophase and is given in a fraction of a month. **A**: Association of lifestyle factors as well as frailty on seasonal patterns. **B**: Whole blood cell counts associated with seasonal patterns

Next, we modelled the sine of the seasonal pattern and computed the association between lifestyle, demographic, and blood cell factors with changes in the amplitude (i.e., the difference between the baseline and the maximum value observed across the year), baseline level (displacement, equivalent to the overall mtDNA abundance corrected for the seasonal pattern) and phase shift (acrophase, i.e., a shift of the wave earlier or later in the year). We found that many factors known to influence mtDNA abundance [10, 35, 42] are still associated with displacement, thus increasing, or decreasing the baseline level of mtDNA abundance (Fig. 3). We found that increased age, BMI and smoking behavior both decreased the observed amplitude, indicating that those factors reduce the natural seasonal variation observed. In contrast, a higher amplitude in mitochondrial abundance was associated with increased eGFR values, implying better kidney health with stronger seasonal changes. In addition, a participant with university degree had a decreased acrophase (i.e., earlier peak), while a higher BMI and lack of physical activity resulted in a later peak. In addition, increased white blood cell count (particularly monocytes, neutrophils and eosinophiles) was correlated to a reduced amplitude. An elevated platelet and lymphocyte count was correlated to increased acrophase (i.e., later peak in the year) and reticulocytes were associated with an earlier peak.

### Diseases and mortality associated with seasonal variations

**Fig. 4:**
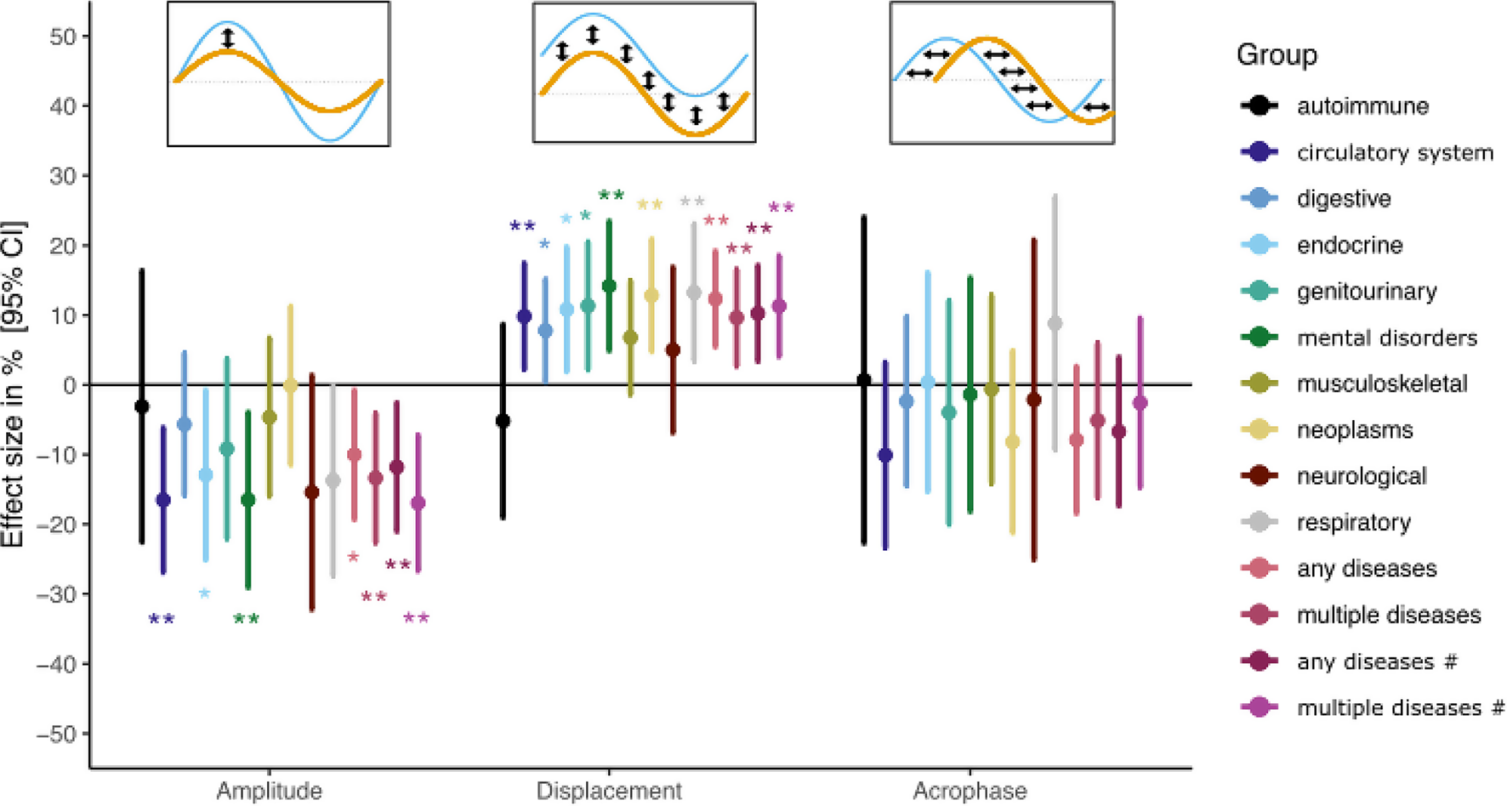
Seasonal variation of mitochondrial abundance associated with incident disease risk. We used linear regression to model the impact of the sine wave on incident disease risk in the UK Biobank. We found that the amplitude and displacement but not acrophase were associated with major incident disease groups. Any disease indicates any of the investigate disease while multiple diseases denote the development of two or more major disease groups. # = all/multiple diseases excluding musculoskeletal and autoimmune diseases and neoplasms. * = *P* < 0.05, ** = *P* < 0.01, *** = *P* < 0.001

In the next step, we aimed to understand whether an individual’s risk to develop a disease or overall mortality could be influenced by seasonal fluctuations in mtDNA abundance. To investigate this, we extracted incident diseases from hospital episode spell data and asked whether individuals that develop a certain disease have an altered amplitude, displacement or acrophase of mitochondrial content across the season (Fig. 4). When we investigated those effects, we found that patients that developed a cardiovascular disease had a 16.5% lower amplitude (95%CI: 6.2%;26.9%, P-value = 0.0018) than those that did not develop such a disease. This means that individuals with a higher risk for cardiovascular disease should have a less pronounced seasonal change with lower values in winter and higher in summer. Similarly, a reduced amplitude was associated with endocrine diseases (12.9%, 95%CI: 0.7%;25.1%, P-value = 0.0379), mental disorders (16.5%, 95%CI: 3.9%;29.1%, P-value = 0.0101) and respiratory disease (13.7%, 95%CI: 0%;27.5%, P-value = 0.05). Overall, individuals with a hospitalization due to any of the investigated diseases had a 10.0% lower amplitude (95%CI: 0.7%;19.3%, P-value = 0.0342), while individuals with multiple diseases had a 13.1% lower amplitude (95%CI: 3.8%;22.5%, P-value = 0.0059). Restricting the analysis to the four disease groups which displayed a significantly different amplitude, the observed effects were increased, as expected (Fig. 4). Generally, higher levels of mitochondrial abundance (after account for seasonal effects) were associated with higher disease risk, in agreement with a recent study [10]. Apart from musculoskeletal, autoimmune, and neurological diseases, we found statistically significant associations at *P* < 0.05 for all other diseases as well as the combined phenotype for any disease (Fig. 4). Importantly, individuals that developed a disease did not show a difference in acrophase.

We also conducted a PheWAS for all diseases occurring at least 500 times during follow-up (640 diseases in total). We found that changes in mtDNA abundance were significantly associated with myeloproliferative disease (OR per S.D.: 1.46 (95% CI: 1.36;1.57), P-value = 2.2 × 10^− 25^) and chronic airway obstruction (OR per S.D.: 1.05 (95% CI: 1.03;1.07), P-value = 1.0 × 10^− 5^). After accounting for multiple testing, no other diseases were associated with amplitude, displacement or acrophase.

Finally, we investigated whether the seasonal pattern is associated with all-cause mortality. We found that individuals which died during the median 7.81 years of follow-up (range: 0.01 years – 15.5 years) had a 22.8% reduced amplitude (95% CI: 5.6%;39.9%, P-value = 0.0009) and no statistically significant difference in the acrophase (1.7% of a month, 95% CI: -22.9%;19.5% of a month, P-value = 0.87). There was also no significant difference observed in the displacement in individuals that died compared to those that did not during follow-up (0.11 S.D. from the mean, 95% CI: -0.008;0.24, P-value = 0.068).

### Metabolomics and proteomics of seasonal MtDNA abundance changes

**Fig. 5:**
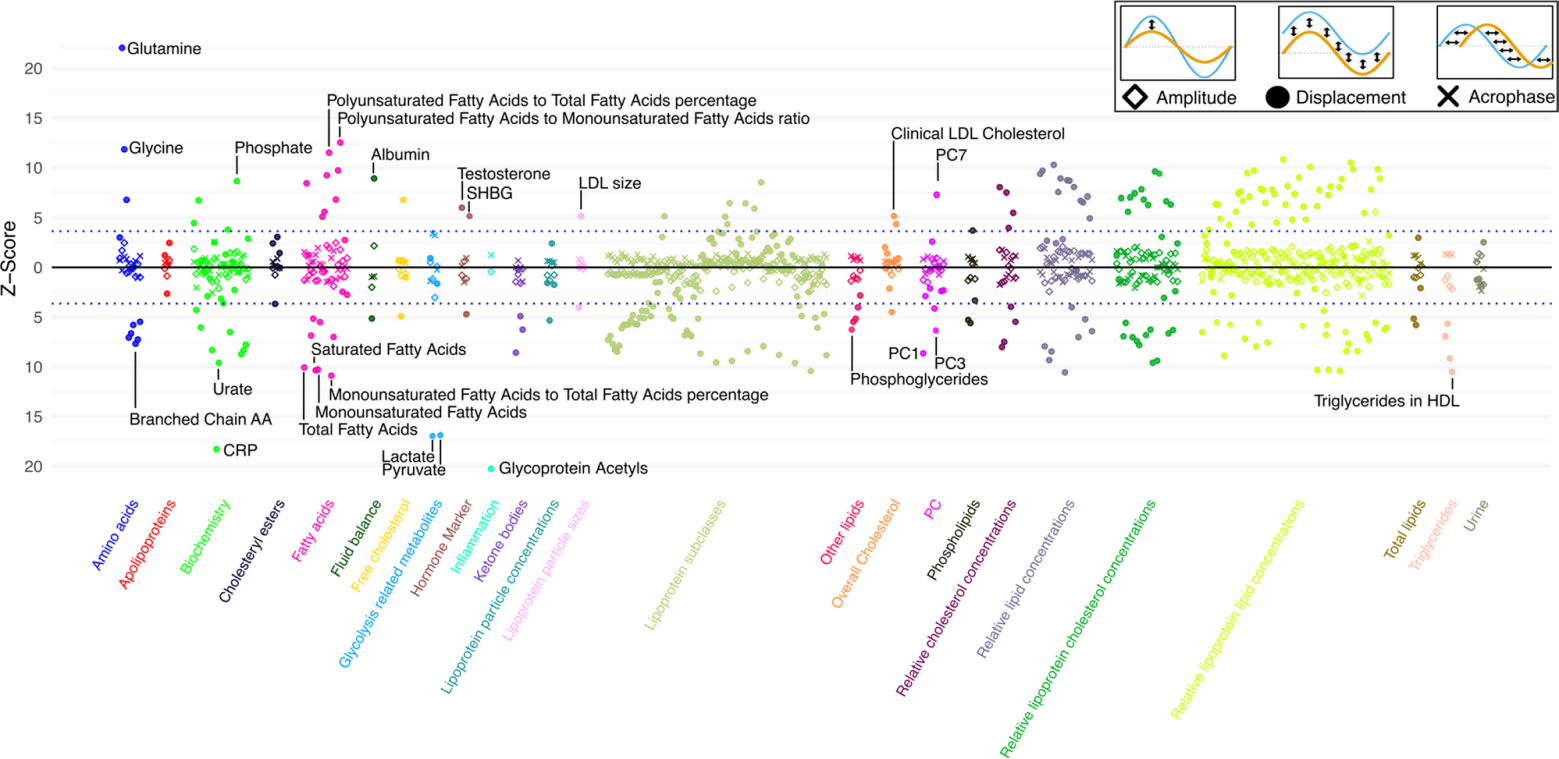
The association between laboratory markers (labWAS) and seasonal pattern of mitochondrial abundance. The association between metabolomic and other blood markers with seasonal mtDNA abundance patterns is shown as a manhattan plot. For each metabolite, we computed a linear regression model with mtDNA abundance as the outcome and modelled the interaction between that marker and the seasonal pattern to extract their effect on amplitude, acrophase and displacement. The analyses were adjusted for lifestyle and immune markers and additionally for total cholesterol to reduce the impact of cholesterol measurement in the results. The horizontal dotted lines denote the Z-Scores corresponding to the Bonferroni corrected P-value threshold of 0.0001 (i.e., 0.05/379 markers). Dots represent the effects observed on displacement, X the effects observed on acrophase and the diamond the effects on the amplitude

**Fig. 6:**
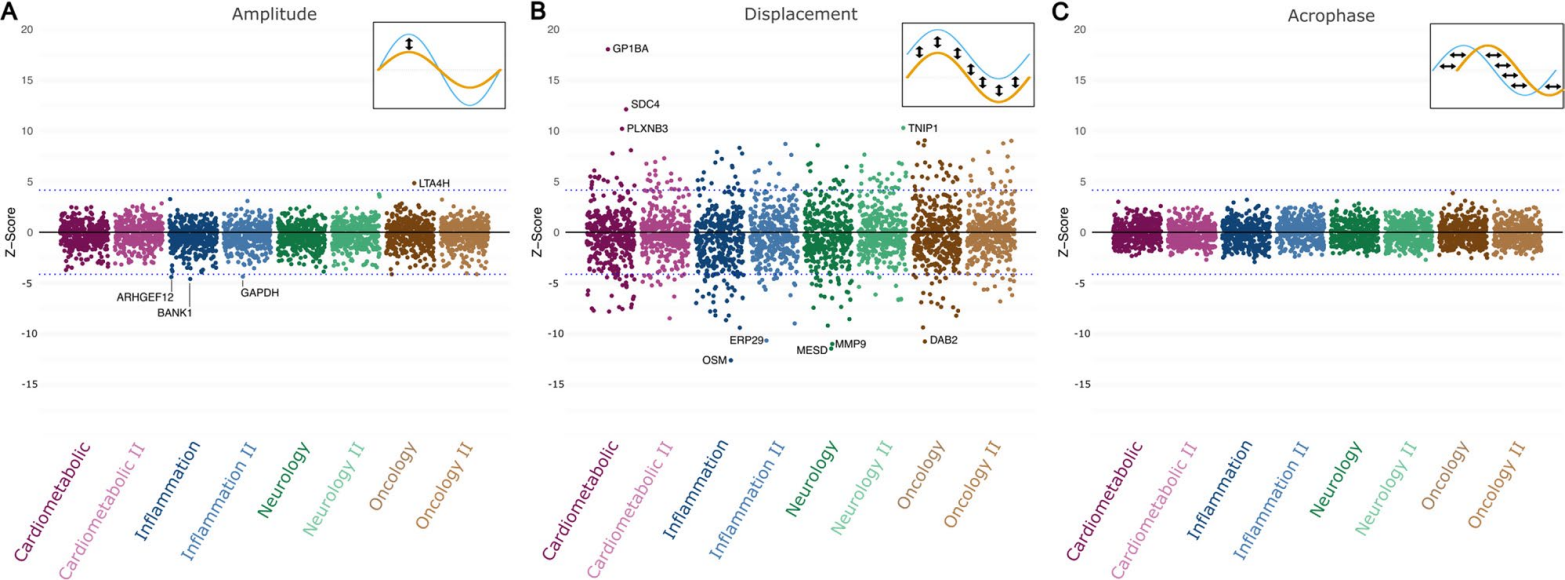
Proteome-wide association study on seasonal patterns of mitochondrial abundance. The correlation between proteins and seasonal patterns of mitochondrial abundance computed with linear regression, adjusted for lifestyle and blood cell markers as well as total cholesterol and the first three principal components computed from the protein data. Each dot represents the Z-Score of the effect on the respective aspect of the seasonal patterns. Positive values indicate that the protein is associated with increased values in the respective pattern, while negative values indicate the opposite. The horizontal dotted lines denote the Z-Scores corresponding to the Bonferroni corrected P-value threshold of 0.000015 (i.e., 0.05/ 2923 markers). Proteins with a Z-Score above 6 or below − 10 are shown in Panel B. **A**: Protein markers associated with mtDNA amplitude (difference between maximum and minim value). **B**: Protein markers Protein markers associated with mtDNA displacement (vertical shift in mtDNA abundance). **C**: Protein markers associated with mtDNA acrophase (horizontal shift across the year)

Building on those results, we next investigated whether there was a correlation between metabolomic or proteomic markers and the seasonal pattern. In the laboratory wide scan (labWAS, Fig. 5 and Supplementary Table 1), we found that the observed Z-Scores were highly correlated between the three seasonal characteristics. We observed that the Z-Scores were negatively correlated between amplitude and acrophase (correlation coefficient − 0.54), acrophase and displacement (correlation coefficient − 0.63) but positively correlated between amplitude and displacement (correlation coefficient 0.72). This indicates that the same markers that influence displacement are potentially also similarly associated with the amplitude. Looking at the markers individually, we found that many metabolomic markers were statistically significantly associated with displacement after correction for multiple testing. However, none of the markers were significantly associated with a shift in acrophase or change in amplitude after Bonferroni correction. Next, we investigated whether seasonal effects are potentially associated with protein measurements (Fig. 6 and Supplementary Table 2). Similar to the results from the metabolomics approach, most proteins were associated with displacement. Nevertheless, we found four proteins statistically significantly associated with amplitude (ARGHEF12, GAPDH, BANK1 and LAT4H).

**Fig. 7:**
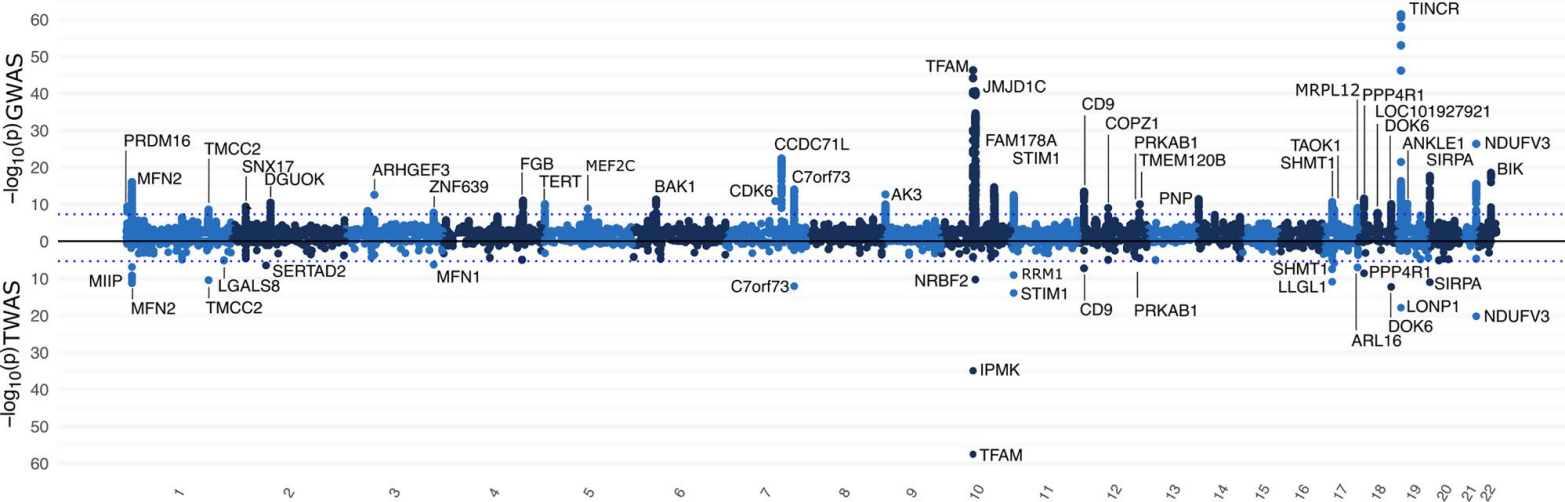
Genome-wide and transcriptome-wide association study results for displacement (overall mtDNA abundance). Top panel (above 0): Genome-wide association study (GWAS); Manhattan plot of 6,112,950 variants with minor allele frequency greater 1% and their association with displacement. Genes closest to the association results are indicated above the association peaks. Bottom panel (below 0): Transcriptome-wide association study (TWAS); Genes expression associated with displacement estimated with TWAS-fusion. Genes below Bonferroni corrected P-Value threshold are labelled excluding duplicate genes. The blue line denotes genome-wide significance (*P* < 5.00 × 10^− 08^, top panel) or transcriptome wide significance (*P* < 1.00 × 10^− 06^). In the GWAS analysis, the closest genes in annotated while in the TWAS the respective gene is indicated

### Genomic dissection of MtDNA seasonal effects

To understand the genetic basis of seasonal effects, we conducted a genome-wide association study modelling the interaction between 6,112,950 common genetic markers and the seasonal pattern in mitochondrial abundance. We observed little evidence for residual population stratification as the genomic inflation factors were 1.00, 1.10 and 1.01 for the GWAS related to amplitude, displacement and acrophase, respectively (Supplementary Fig. 1). The pairwise correlation coefficient of the Z-Scores observed between the GWAS for amplitude and displacement was 0.11, indicating that genetic factors that increase baseline mtDNA abundance also influence, on average, amplitude in the same orientation. In contrast, we found no correlation between the Z-Scores observed for amplitude and acrophase (correlation coefficient = 0.00) and a negative correlation between acrophase and displacement (correlation coefficient = -0.086). First, we investigated whether our GWAS overlaps with a recent GWAS by Gupta et al. [10]. The correlation coefficient between the betas for displacement observed in our study and the GWAS by Gupta et al. was 0.98, indicating excellent overlap. Out of 2754 genome-wide significant variants from Gupta et al., 1348 were also genome-wide significant in our sample and only 16 did not reach significance. Those 16 variants did not show a statistically significant association with amplitude or acrophase, however. In total, we found 36 loci associated with displacement (i.e., overall mitochondrial abundance) that reached genome-wide significance (Fig. 7 and Supplementary Table 3). 24 of those loci were identified by Gupta et al. with genome-wide significance, while 10 were at least nominally significantly associated at *P* < 0.05. One locus (rs58105891 in TMCC2) is novel and did not reveal associations in prior GWAS.

However, there were no genome-wide significant regions associated with acrophase or amplitude. We found two loci with suggestive evidence associated with amplitude (rs4408948 on chromosome 4, P-value = 1.04 × 10^− 07^ and rs145637468 on chromosome 9, P-value = 3.71 × 10^− 07^) and one locus associated with acrophase (rs76900225 on chromosome 8, P-value = 1.26 × 10^− 07^). We estimated the heritability of displacement to be 2.1% (95% CI: 1.67%; 2.53%). In contrast, we were not able to compute heritability estimates for displacement or acrophase due to the lack of genome-wide significant findings. The resulting summary statistics from each GWAS were used to compute a transcriptome-wide association study. While we did find 29 genes significantly associated with displacement (Fig. 6 and Supplementary Table 4), none remained after accounting for multiple testing in the TWAS for amplitude or acrophase.

## Discussion

In this study, we aimed to provide insights into the epidemiological basis of seasonal mtDNA oscillation. Our results indicate that age, BMI, smoking behavior as well as white blood cell count reduce the amplitude of the seasonal pattern and thus the difference between maximum and minim values observed across the year. Similarly, BMI, lack of physical activity, a higher education as well as platelet, lymphocyte and reticulocyte count influence the acrophase and therefore shift the whole seasonal pattern to earlier or later peaks in the year. However, the strongest effects were generally observed for the displacement, i.e., the vertical shift in the sine wave across the season, which is equivalent to the overall mtDNA abundance, accounting for the seasonal pattern. This was true for the lifestyle, demographic, immune and red blood cell counts, the labWAS, the protein-wide, genome-wide, and transcriptome-wide association study. Nevertheless, we observed that individuals that developed a disease or died due to any cause had a significant change in the amplitude and not in acrophase or displacement, which warrants further investigation.

The observed variation in mtDNA abundance across the year was estimated from the reads mapping to the mitochondrial genome and normalized by total read count. We also saw the same effect when using our previously established method to estimate mtDNA from genotyping arrays [35]. In addition, a recent study using whole genome sequencing [10] also reported this effect, indicating that it is unlikely to be a technical artifact. The observed effect was also stable across all three years of recruitment in the UKB and also when adjusting for potential batch effects such as WES release tranche, recruitment center and year of recruitment. Nevertheless, while we can model the seasonal changes as a sine wave, we do not know the consequence of altered mtDNA abundance in summer or winter. For instance, an individual with higher-than-expected mtDNA abundance in winter might also have higher mtDNA abundance in summer (i.e., a generally increased mtDNA amount) or might have a higher amplitude instead. To study this, longitudinal mtDNA measurements would be necessary, which are usually not available for most studies since genotyping (with arrays or sequencing) is usually only performed once per patient. The observed amplitude is similar in effect size as the difference observed between males and females, thus the total variation across the season is more than twice that effect. This indicates that the seasonal variation is an important factor in governing total mtDNA abundance in individuals, which is why we performed several analyses to investigate this phenomenon. However, our studies are only done with one measurement per person, so the true seasonal effects might be more different than the ones we observed here.

Several demographic and lifestyle factors influence the seasonal pattern. While most factors influence the displacement and thus the vertical shift of the distribution as was previously found by other studies [10, 35, 42], there were fewer associations for amplitude and acrophase. Generally, less healthy behavior such as a higher BMI and increased pack-years as well as increased age seemed to decrease the amplitude of mtDNA abundance resulting in lower maximum and higher minimum seasonal values. This observation fits the results obtained from the disease associations where individuals that developed diseases generally had lower amplitudes than individuals that remained healthy. The precise biological mechanisms behind the observed disease associations, are, however difficult as only few markers were associated with seasonal patterns such as amplitude and acrophase. In this study, we observed that higher white blood cell counts also correlated to a reduced amplitude. Previous studies showed that increased white blood cell count increased the risk to develop a cardiovascular disease [43]. However, our PheWAS was adjusted for white blood cell count as well as BMI and pack-years (which showed a similar pattern as white blood cell count) and other risk factors for cardiovascular disease. Thus the observed association is likely independent of those risk factors and unlikely to work through modulation of those risk factors. Indeed, we observed only minor attenuation of the association between classical risk factors and cardiovascular disease when adjusting for mitochondrial abundance or season (data not shown).

Similar to the amplitude, we found that several lifestyle and demographic factors influenced acrophase. The pattern observed here, however, is less clear. Generally considered disease risk factors (such as obesity, lack of physical activity, and less education) had differential effects on acrophase. This might also explain why we did not observe an association between acrophase and incident diseases. This is also in line with the observation that an increased frailty index was not associated with acrophase. We performed the disease association analyses first on major disease groups to increase our power since our method relies on computing interaction terms between the sine and cosine function of the seasonal pattern. Thus, our power was reduced, resulting in few findings investigating all 640 common diseases when accounting for multiple testing. Further studies, including additional cohorts modelling the seasonal pattern would be necessary to identify which specific diseases in each disease group are responsible for the observed associations. Importantly, changes in the amplitude observed at baseline were associated with increased all-cause mortality. This observation is in line with the results obtained from the disease associations and points towards mechanisms that could be useful targets for treatment. However, whether the observed effect on mortality is causal remains to be investigated, potentially in a propensity matched cohort. In addition, the observed effects should also be replicated in an independent cohort to assess whether the recruitment strategy could influence the observed effects. In addition, replication of our results in individuals from different ethnicities as well as populations living in other climates (such as equatorial regions without seasonal variation as well as individuals from the southern hemisphere with inverted seasons) is warranted to investigate the generalizability of our findings.

While we found that changes in the amplitude of mtDNA across the season were a risk factor for cardiovascular, endocrine, and respiratory diseases as well as mental disorders, we found little molecular markers that would explain those associations. Neither the labWAS, nor the genomic approach found statistically significant markers associated with amplitude or acrophase. Part of the explanation could be the reduced power due to the requirement to model two interaction terms (one with the sine and one with the cosine function) in the association testing. In addition, the mtDNA abundance overall only has a small heritability, therefore limiting the power to identify markers associated with the trait in an interaction analysis. Furthermore, there is a large degree of interindividual variability, so the association results could be confounded by individual variation across the season that cannot be accurately modelled without additional markers and longitudinal data. Although the strongest effects in the proteomic approach were also observed for displacement, we found several proteins to be associated with mitochondrial amplitude. Those genes are largely involved in regulating parts of the adaptive and innate immune systems: ARHGEF12 (Rho Guanine Nucleotide Exchange Factor 12, also known as LARG, Leukemia-associated RhoGEF) is a protein involved in the regulation of RhoA GTPAse and is likely involved in immune cell function [44]. In addition, several studies found association signals near this gene for monocyte count. BANK1 (B Cell Scaffold Protein With Ankyrin Repeats 1) is a B-cell specific scaffold protein that is likely involved in autoimmune diseases [45]. Similarly, we found that increased GAPDH levels in plasma resulted in reduced mtDNA amplitude. This protein has many functions in the cell such metabolism, adhesion, and, regulation of transcription [46] but has also been found elevated in plasma in patients with liver cirrhosis [47], Alzheimer’s disease [48] and is likely involved in immune cell function [49]. Likewise, Leukotriene A4 Hydrolase (LTA4H) is an enzyme that can degrade proline-glycine-proline (PGP), which is a neutrophil attractant. Thus, those proteins might be associated with the amplitude, since we also observed that differences in white blood cell counts to be similarly affected by changes in the mtDNA amplitude. Importantly, we adjusted for blood cell count in the proteomic analyses in order to not confound our analyses by those factors. Since we observed the association with those immune related proteins anyways, this indicates that they likely act independently of blood cell count. The precise mechanism behind the observed association between mtDNA seasonal amplitude and plasma levels of those proteins is, therefore, not easily described and requires additional research.

Our results have important implications for future research. Previous studies often reported conflicting results regarding the association between mtDNA abundance and diseases. While many of those issues can be attributed to inadequate adjustment for confounders (see [10]), part of the issue could also be due to seasonal effects, which could confound association analyses. While we found several associations with overall mtDNA abundance (displacement), most of the diseases were also associated with changes in the amplitude. Similarly, changes in amplitude were associated with all-cause mortality while acrophase and displacement were not. Thus, accounting for season and potentially the amplitude would be useful when investigating disease associations in future studies.

In our analyses, we imputed missing values to the median (for BMI, packyears smoked and eGFR) or using collinearity in the data (proteomics data). In a sensitivity analysis using non-imputed data, we found virtually the same results, which was expected as the missingness of the data is rather low. The choice to perform imputation was to increase our power, which was necessary to investigate the interaction between markers and seasonal parameter. In addition, we excluded individuals with extremely high or low mtDNA abundance measurements. In total, 9650 individuals were excluded due to extreme mtDNA measurements (i.e., more than 4 times the interquartile range from the median). Those extreme values could be a result of technical issues with the enrichment (i.e. low genomic coverage or very high mtDNA coverage due to stronger non-specific mtDNA adherence to the beads) or due to biological variation. Since those individuals represent only around 2% of the population but have (comparably) extreme mtDNA abundance estimates, they could skew the results, particularly if they were recruited in a similar season, for instance.

In our analyses, we adjusted for many factors that could explain this effect by exhibiting seasonal effects (such as blood cell count [50] or vitamin D levels [51]), be influences by seasonal effects (such as smoking behavior [52], alcohol consumption [53] or exercise) as well as technical variables (such as year and hour of recruitment, study center and thus latitude of recruitment). Since the seasonal pattern was evident with those adjustments, this therefore strongly suggests that the seasonal pattern has other causes independent of those factors. Recent reports indicate that many blood parameters such as red blood cell count also fluctuate in a similar pattern observed here for mtDNA [54, 55], which could be attributed to seasonal differences in atmospheric pressure and thus oxygen supply [56]. Thus, the pattern observed for mtDNA abundance could be an evolutionary adaptation to the seasonal availability of oxygen. However, we did not observe that the amplitude of mtDNA abundance influences red blood cell parameters and there are mixed effects observed for displacement i.e., overall mtDNA abundance. Therefore, the precise role of seasonal effects in mtDNA abundance in adaptation and evolution is not clear. While such seasonal effects for mitochondria have been observed in hibernating mammals, its role in non-hibernating species is unknown. One of the important functions of mitochondria is the production of heat. Since mitochondria maintain a temperature roughly 15 °C above their environment, their precise control could be crucial to maintain adequate body temperature throughout the year [57]. Finally, the mitochondrial seasonal pattern could be due to an adaptation of humans to changing seasons and thus changes in the scarcity of food, which could have been necessary after the great expansion event. This would fit the observation that a higher body mass index (i.e., surplus of caloric intake) is correlated to a reduced amplitude. However, the results could also point towards a different explanation: Maladaptation of the seasonal mtDNA abundance to a modern lifestyle could lead to increased weight gain (e.g., a higher amplitude could result in a more efficient fat storage), thus resulting in a similar association pattern. However, due to limited longitudinal data, we can only speculate on the precise mechanisms behind the observed associations. The change in mitochondrial abundance could also be related to a mechanism to recycle mitochondria which have accrued damaging mutations. Previous studies found that highly proliferative tissues have a lower heteroplasmy compared to low-turnover tissues [58, 59]. Thus a faster turnover of immune cells between seasons could be beneficial to reduce heteroplasmy burden over many years. However, since the life-span of cell carrying mitochondria in blood is much shorter than a seasonal interval (in the range of a few days generally [60]), it is much more likely that cells with damaged mitochondria are themselves replaced instead of the mitochondria within those cells. Therefore, we speculate that the mechanism is an adaptation to differences in diet, temperature [57], atmospheric pressure [61], or light exposure which differs between seasons. Interestingly, the peak and trough of mitochondrial seasonal oscillation roughly coincides with winter and summer solstice, respectively. This strong tie with exposure to overall daylight duration, air pressure or temperature [62] could therefore be mechanisms driving changes in mitochondrial abundance, even in non-hibernating mammals such as humans. While out of scope, we would thus hypothesize that a similar trend observed here in the UK Biobank should present in humans living in the southern hemisphere as well, albeit with reverse pattern i.e., the highest mtDNA abundance estimates observed in the summer months of the northern hemisphere. In contrast, in regions with less severe climatic differences between the seasons, the mtDNA seasonal pattern should also be attenuated with a weaker amplitude.

Taken together our results show that seasonal changes in mitochondrial abundance are a risk factor for multiple major diseases as well as overall mortality in our aging society. The precise control of the seasonal pattern is, however, elusive, and likely not influenced by common genetics, metabolite levels or circulating proteins. Further research is therefore warranted, particularly with longitudinal DNA sampling and mtDNA abundance estimation.

## Electronic supplementary material

Below is the link to the electronic supplementary material.

Supplementary Material 1: Table 1: Association results from the labWAS. Table 2: Statistically significant markers identified in the proteome-wide scan. Table 3: Statistically significant markers identified in the GWAS for displacement.

Supplementary Material 2: qq-plot of the genome-wide association studies. The expected P-values was plotted against the observed P-values on a negative decadic logarithm scale. Panels from left to right: qqplot for amplitude, displacement and acrophase. The observed genomic inflation factors were 1.00, 1.01 and 1.10 for amplitude, acrophase and displacement, respectively.

## Data Availability

Access to phenotypes, biospecimen and genotypes from the UK Biobank can be requested from https://biobank.ndph.ox.ac.uk/. The summary statistics from the GWAS will be deposited at the NHGRI-EBI GWAS Catalog. The analyses scripts will be published on the group’s GitHub page: https://github.com/GrassmannLab. The raw and normalized (quality-controlled) mtDNA abundance estimates will be returned to the UK Biobank for further dissemination.

## Glossary

Displacement: Shift in the seasonal pattern of mtDNA abundance in a vertical orientation (Fig. 1**B**). This is equivalent to overall changes of mtDNA abundance, which previous studies investigated.
Acrophase: Shift in the seasonal pattern of mtDNA abundance to earlier or later peak values (Fig. 1**B**). In particular, factors influencing acrophase influence the peaks to occur earlier or later in the year.
Amplitude: Change in the height of the peak in the seasonal pattern (Fig. 1**B**). Factors influencing the amplitude result in higher or lower peaks, but the mean of the distribution does not necessarily change.
Marginal effect: In the interaction analysis (Fig. 1**C**), the change in the amplitude and acrophase due to certain markers is indicated by the effect size of the interaction term. In addition to the interaction term, the marginal effect of the marker can also be retrieved in the model. The marginal effect is equivalent to the displacement of the mtDNA abundance due to the factor, adjusted for the seasonal pattern.
