# Supplementary material for: Tapping natures rhythm: the role of season in mitochondrial function and genetics in the UK biobank
Source: Hum Genomics. 2025 Mar 29;19:34. doi: 10.1186/s40246-025-00743-8 (PMC11954186; doi:10.1186/s40246-025-00743-8)
Supplement: Supplementary file 2 — Supplementary Material 2: qq-plot of the genome-wide association studies. The expected P-values was plotted against the observed P-values on a negative decadic logarithm scale. Panels from left to right: qqplot for amplitude, displacement and acrophase. The observed genomic inflation factors were 1.00, 1.01 and 1.10 for amplitude, acrophase and displacement, respectively. [file 40246_2025_743_MOESM2_ESM.docx]

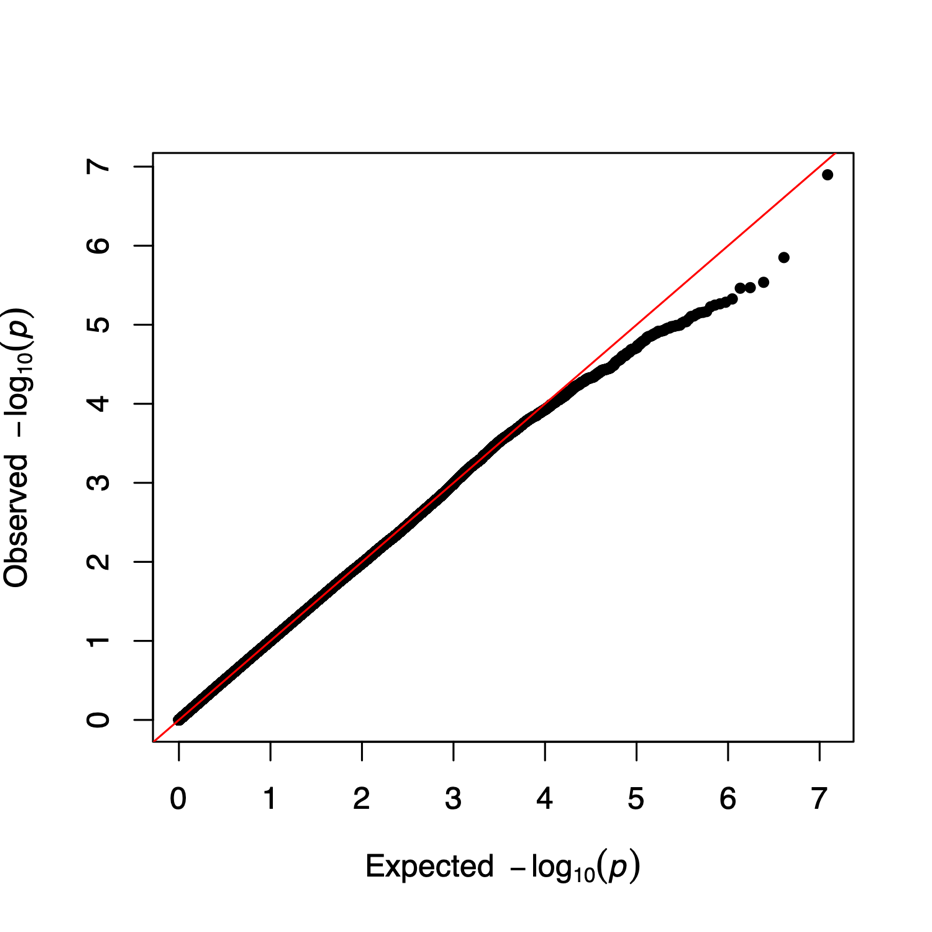

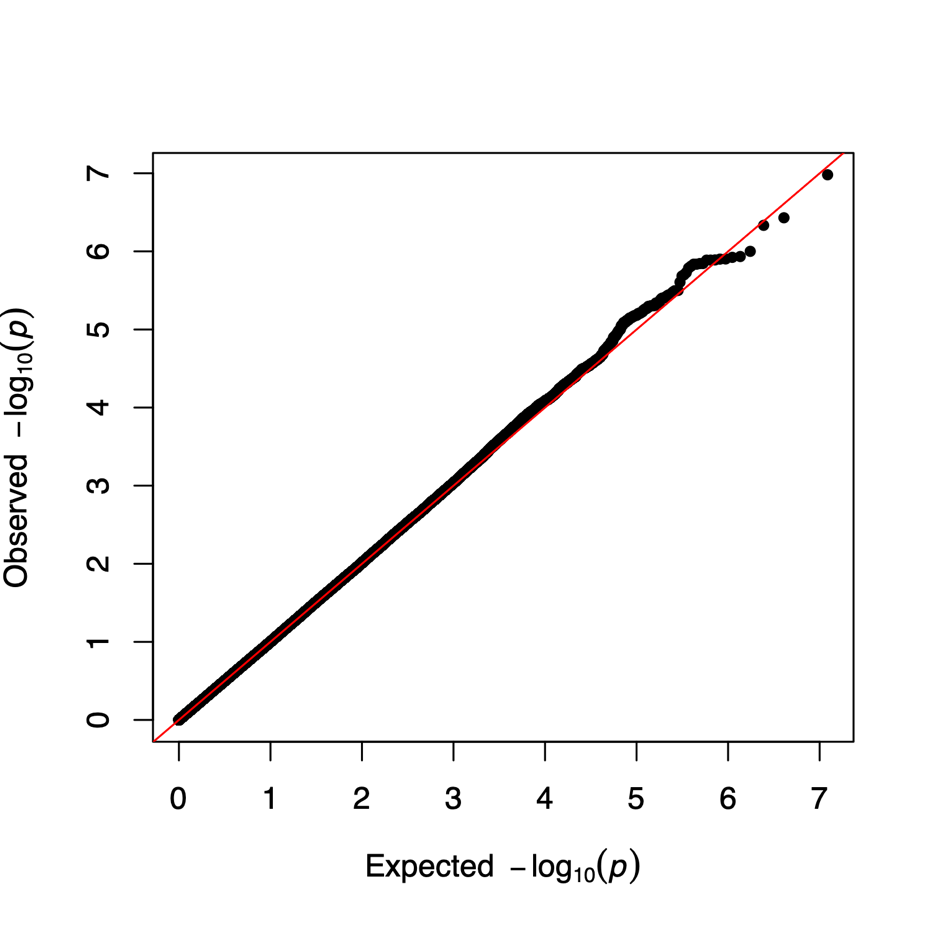

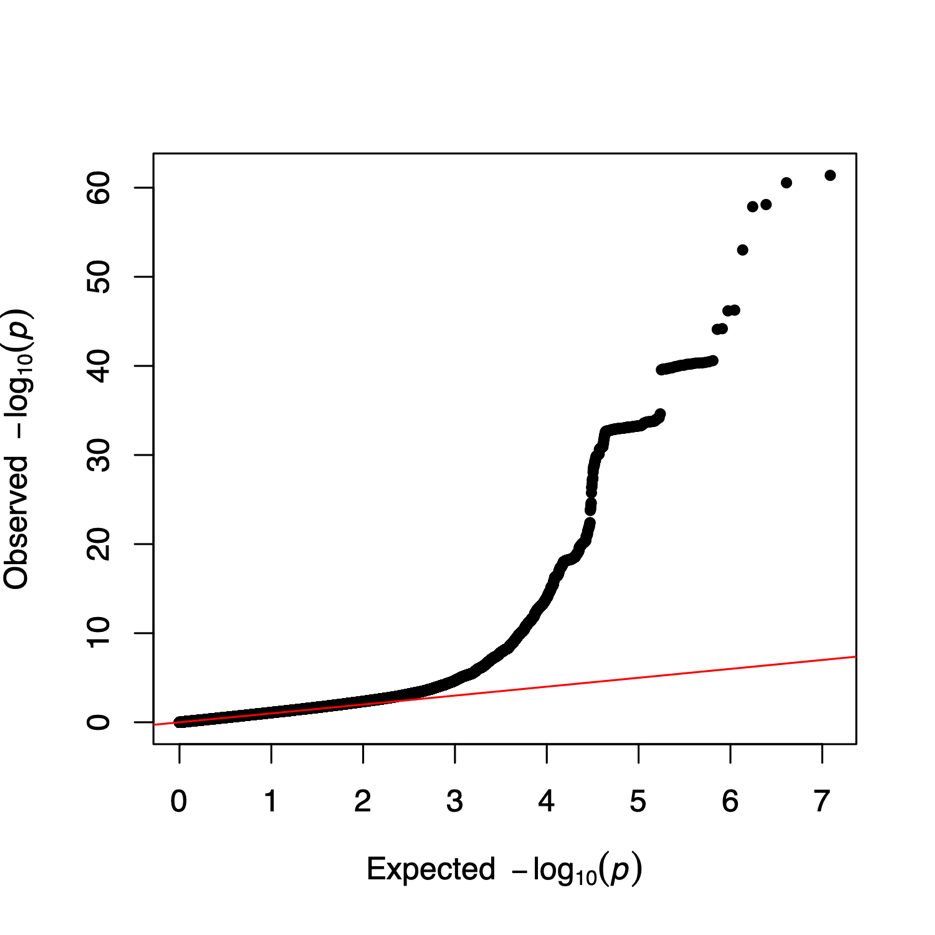


**Supplementary Figure 1. qq-plot of the genome-wide association studies.** The expected P-values was plotted against the observed P-values on a negative decadic logarithm scale. Panels from left to right: qqplot for amplitude, displacement and acrophase. The observed genomic inflation factors were 1.00, 1.01 and 1.10 for amplitude, acrophase and displacement, respectively.
